# Cytotoxic Sesterterpenoids Isolated from the Marine Sponge *Scalarispongia* sp.

**DOI:** 10.3390/ijms151120045

**Published:** 2014-11-04

**Authors:** Yeon-Ju Lee, Jeong-Woo Lee, Dong-Geun Lee, Hyi-Seung Lee, Jong Soon Kang, Jieun Yun

**Affiliations:** 1 Marine Natural Product Chemistry Laboratory, Korea Institute of Ocean Science and Technology, Ansan 426-744, Korea; E-Mails: toyjwlee@gmail.com (J.-W.L.); trchapman@hanmail.net (D.-G.L.); hslee@kiost.ac (H.-S.L.); 2 Bio-Evaluation Center, Korea Research Institute of Bioscience and Biotechnology, Cheongwon 323-833, Korea; E-Mails: kanjon@kribb.re.kr (J.S.K.); jyun@kribb.re.kr (J.Y.)

**Keywords:** sponge, Scalarispongia, sesterterpenoid, scalarane, cytotoxicity

## Abstract

Eight scalarane sesterterpenoids, including four new compounds, were isolated from the marine sponge *Scalarispongia* sp. The structures of the new compounds were elucidated by 2D-NMR and HRMS analyses. All of the isolated compounds, with the exception of 16-*O*-deacetyl-12,16-*epi*-scalarolbutanolide, showed significant *in vitro* cytotoxicity (GI_50_ values down to 5.2 μM) against six human cancer cell lines.

## 1. Introduction

Scalaranes are a group of sesterterpenoids with a characteristic carbon skeleton consisting of four cyclohexane rings joined together, and an optional five-membered heterocycle, which contains an oxygen or a nitrogen [1]. These compounds have been exclusively found from marine organisms such as sponges and nudibranchs, and never been isolated from terrestrial organisms, whereas a few examples of other types of sesterterpenoids isolated from plants, such as picracin [2,3], leucosceptrine [4,5], and salvimirzacolide [6], have been reported. It is believed that scalaranes play a key role in the chemical defense of marine invertebrates, as a large majority of these compounds exhibit cytotoxic and antifeedant activity. Thus, scalaranes have been considered as chemotaxonomic markers in marine invertebrates, and suggested as potential lead compounds for therapeutic agents.

Since the isolation of the first scalarane compound (scalarin, **1**) from marine sponge *Scalarispongia scalaris* (previously known as *Cacospongia scalaris*) [7], extensive studies about the chemicals contained in marine sponges of *Scalarispongia* species have led to the discovery of various scalarane sesterterpenoids [8,9,10,11,12,13], and other terpenes such as furanosesterterpenes [14] and furanoditerpenes [15,16].

Following our research on cytotoxic compounds obtained from Korean marine sponges, we herein report eight scalarne sesterterpenoids (**1**‒**8**), including four new compounds (**5**‒**8**), exhibiting promising level of cytotoxicity, which were isolated from *Scalarispongia* sp. collected off the coast of Dokdo, Republic of Korea.

## 2. Results and Discussion

### 2.1. Isolation of Scalarane Sesterterpenoids from a Scalarispongia sp. Marine Sponge

A freeze-dried *Scalarispongia* sp. sponge was macerated and extracted with methanol and dichloromethane; the combined extract was partitioned between *n*-butanol and water. The *n*-butanol fraction was then partitioned between 15% aqueous methanol and *n*-hexane, and the 15% aqueous methanol fraction was further partitioned between dichloromethane and 50% aqueous methanol. The dichloromethane fraction was subjected to silica column chromatography, followed by HPLC using a silica column, to afford eight scalarane compounds (**1**‒**8**).

### 2.2. Structure Elucidation of Isolated Compounds

**Figure 1:**
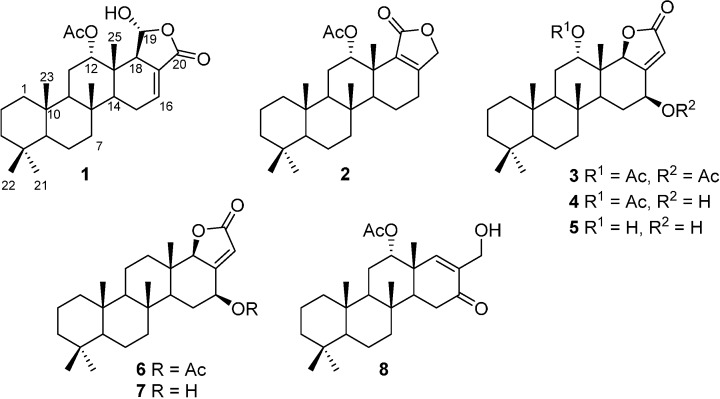
Structures of the isolated scalarane sesterterpenoids.

A comparison of our NMR, LRMS and optical rotation data with those reported in previous literatures confirmed that compounds **1**‒**4** are scalarin [7], 12-*epi*-12-*O*-acetylscalarolide [12], 12-*O*-acetyl-12,16-*epi*-scalarolbutenolide, and a 16-*O*-deacetylated derivative of **3** [17], respectively (Figure 1).

**Figure 2:**
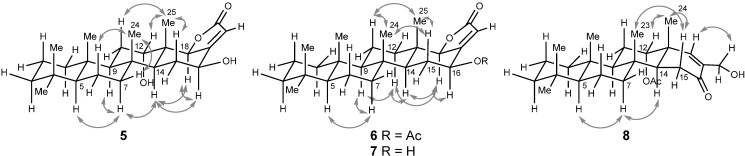
Selected NOESY correlations for compounds **5**‒**8**.

Compounds **3**‒**5** share the same carbon and oxygen framework with the only difference between these compounds being the degree of acetylation, as judged by ^1^H- and ^13^C-NMR (Table 1) and HRMS data. In particular, compound **5** was identified as 16-*O*-deacetyl-12,16-*epi*-scalarolbutanolide (Figure 1), in agreement with the molecular formula of C_25_H_38_O_4_ obtained by HRMS analysis. While such a compound has never been reported, its C-12 epimer has been isolated from *Hyrtios erecta* [18]. The previously reported ^1^H-NMR data for the C-12 epimer showed the proton being attached to C-12 (δ 3.67) with a trans-diaxial coupling (*J* = 11.2 Hz) with an axial proton at C-11; no such coupling was observed in the ^1^H-NMR spectra of **5**. The stereochemistry of **5** was further confirmed by NOESY correlations (Figure 2).

**Table 1:** ^1^H- and ^13^C-NMR data (500 and 125 MHz) for compounds **5**‒**7**.

Position	5 *^a^*		6 *^a^*		7 *^b^*	
	δ_C_, type *^c^*	δ_H_ (*J* in Hz)	δ_C_, type *^c^*	δ_H_ (*J* in Hz)	δ_C_, type *^c^*	δ_H_ (*J* in Hz)
1	39.2, CH_2_	1.64, m	40.1, CH_2_	1.68, m	40.2, CH_2_	1.69, m
		0.72, m		0.75, ddd (12.5, 3.5, 3.5)		0.78, ddd (12.5, 3.5, 3.5)
2	18.1, CH_2_	1.42, m	18.8, CH_2_	1.57, m	18.8, CH_2_	1.55, m
		1.22, m		1.40, m		1.40, m
3	41.7, CH_2_	1.20, m	42.2, CH_2_	1.36, m	42.2, CH_2_	1.35, m
		0.98, ddd (13.0,13.0, 3.5)		0.95, ddd (12.5, 12.5, 3.5)		1.10, ddd (13.0, 13.0, 3.5)
4	32.8, C		33.5, C		33.5, C	
5	56.1, CH	0.76, brd (13.0)	56.6, CH	0.82, m	56.7, CH	0.78, m
6	18.2, CH_2_	1.40, m	18.4, CH_2_	1.54, m	18.5, CH_2_	1.54, m
		1.30, m		1.29, m		1.40, m
7	41.7, CH_2_	1.66, ddd, (12.5, 3.0, 3.0)	42.5, CH_2_	1.77, ddd (13.0, 4.0, 3.0)	42.6, CH_2_	1.81, ddd (12.5, 3.0, 3.0)
		0.92, ddd, (12.5,12.5, 3.0)		1.02, ddd (13.0, 13.0, 4.0)		1.00, (12.5, 12.5, 3.0)
8	37.7, C		38.2, C		38.2, C	
9	50.6, CH	1.32, m	61.4, CH	0.87, m	61.4, CH	0.81, m
10	36.6, C		37.8, C		37.8, C	
11	25.2, CH_2_	1.54, m	17.2, CH_2_	1.60, m	17.2, CH_2_	1.59, m
		1.28, m		1.38, m		1.33, m
12	70.2, CH	3.60, dd (3.0, 3.0)	40.5, CH	2.03, m	40.7, CH	2.03, dd (10.0, 3.0)
				1.33, m		1.34, m
13	45.5, C		41.6, C		41.6, C	
14	45.1, CH	1.19, m	51.6, CH	1.09, m	51.9, CH	1.05, m
15	30.6, CH_2_	1.99, ddd (12.5, 8.0, 2.0)	28.0, CH_2_	2.19, ddd (12.5, 7.0, 2.0)	31.7, CH_2_	2.17, ddd (12.5, 7.0, 2.0)
		1.33, m		1.47, m		1.45, m
16	67.4, CH	4.26, dd (10.0, 8.0)	69.8, CH	5.52, ddd (11.5, 7.0, 2.0)	69.0, CH_2_	4.49, ddd (9.3, 7.0, 2.0)
17	175.5, C		167.0, C		171.7, C	
18	83.3, CH	5.06, brs	90.0, CH	4.33, d (2.0)	90.1, C	4.29, brs
19	174.9, C		173.0, C		173.5, C	
20	110.6, CH	5.73, brs	112.4, CH	5.76, dd (2.0, 2.0)	111.9, CH_2_	5.90, brs
21	32.9, CH_3_	0.67, s	33.5, CH_3_	0.82, s	33.4, CH_3_	0.83, s
22	20.8, CH_3_	0.65, s	21.5, CH_3_	0.78, s	21.5, CH_3_	0.79, s
23	16.0, CH_3_	0.68, s	16.5, CH_3_	0.81, s	16.5, CH_3_	0.81, s
24	16.7, CH_3_	0.72, s	17.7, CH_3_	0.85, s	17.7, CH_3_	0.86, s
25	11.6, CH_3_	0.51, s	12.3, CH_3_	0.70, s	12.4, CH_3_	0.69, s
16-OAc			170.0, C			
			21.1, CH_3_	2.16, s		

*^a^* The ^1^H- and ^13^C-NMR spectra are measured in CDCl_3_/CD_3_OD (3:1); *^b^* The ^1^H- and ^13^C-NMR spectra are measured in CDCl_3_; *^c^* Carbons correlating with the corresponding proton.

**Figure 3:**
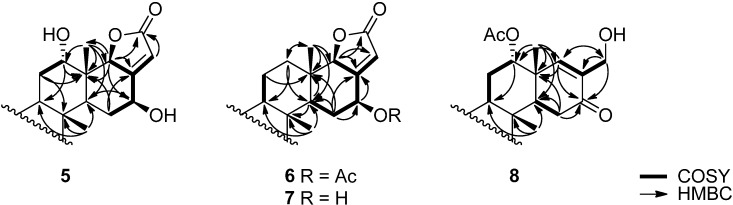
Selected COSY and HMBC correlations for compounds **5**‒**8**.

Compound **6** was deduced to have the molecular formula C_27_H_40_O_4_ based on the analysis of its HRFABMS. In ^1^H- and ^13^C-NMR spectra of **6**, unlike those of **3**, no signals related to oxymethines were detected. Instead, methylene protons at δ 2.03 and 1.33, which shows HSQC correlation with a carbon signal at δ 40.5 and HMBC correlations with carbon signals at (C-25), 61.4 (C-9), and 51.6 (C-14), were observed (Table 1, Figure 3). These findings suggest that no substituent is present at C-12. The stereochemistry at C-16, where the remaining acetate group is attached, was confirmed by NOESY correlations (Figure 2).

Compound **7** was identified as a 16-*O*-deacetylated derivative of **6**, as ^1^H- and ^13^C-NMR data for **7** were similar to those obtained for **6**, except for the signals of the acetyl group (Table 1). This is in agreement with the molecular formula C_25_H_38_O_3_ obtained by the analysis of HRFABMS. While several scalarin and scalaradial derivatives lacking the C-12 oxygen have been reported [19,20,21,22], isoscalarane derivatives such as **6** and **7** have never been reported previously.

The molecular formula of compound **8**, obtained by HRFABMS, was C_26_H_40_O_4_. The carbon signals at δ 202.0, 135.4, and 155.3 as well as a proton signal at δ 6.51, which shows a HSQC correlation with a carbon signal at 155.3, suggested the presence of an α, β-unsaturated ketone. A proton signal at δ 4.16, which shows a HSQC correlation with a carbon signal at 62.1 and HMBC correlations with carbon signals of aforementioned unsaturated ketone, indicated that a hydroxymethyl group is attached to the α-position of the carbonyl group. Based on these observations as well as the NOESY and HMBC correlations displayed in Figure 2 and Figure 3, the structure of **8** could be elucidated unambiguously. Interestingly, most of tetracyclic scalaranes possess an oxo group at C-19 as well as a hydroxy or an acetate group at C-16 [1]. Despite the same oxidation state, the structure of **6** is diametrically opposed to other tetracyclic scalaranes.

The isolated compounds were tested for *in vitro* cytotoxicity against a panel of human cancer cell lines and the results are summarized in Table 2. All compounds, except for **5**, showed potent inhibition of cancer cell growth. Compounds **5** showed no activity even at a concentration of 60 μM. This suggests that substitution at C-12 is not a requirement for cytotoxicity; it is highly probable that the presence of a hydrogen bond donor at C-12 can significantly decrease the cytotoxicity of the scalarane derivatives.

**Table 2:** Growth inhibition of compounds **1**–**8** against a panel of human tumor cell lines *^a^*.

Compound	Cell Line (GI_50_ μM) *^b^*					
	HCT-15	NCI-H23	ACHN	MDA-MB-231	NUGC-3	PC-3
**1**	7.38 ± 0.07	11.0 ± 0.12	6.01 ± 0.02	6.17 ± 0.02	7.25 ± 0.04	5.45 ± 0.02
**2**	6.52 ± 0.05	6.20 ± 0.09	5.43 ± 0.01	6.99 ± 0.03	6.13 ± 0.03	5.36 ± 0.03
**3**	7.75 ± 0.08	5.66 ± 0.09	6.17 ± 0.04	5.41 ± 0.02	5.66 ± 0.03	5.70 ± 0.02
**4**	8.17 ± 0.06	9.02 ± 0.10	6.99 ± 0.02	5.19 ± 0.03	8.98 ± 0.04	7.01 ± 0.02
**5**	>60.0	>60.0	>60.0	>60.0	>60.0	>60.0
**6**	9.11 ± 0.09	21.0 ± 0.09	6.69 ± 0.04	11.3 ± 0.08	21.0 ± 0.02	10.0 ± 0.08
**7**	11.6 ± 0.09	12.2 ± 0.10	7.22 ± 0.07	5.24 ± 0.04	12.2 ± 0.04	6.53 ± 0.03
**8**	7.85 ± 0.05	10.6 ± 0.07	6.60 ± 0.03	9.10 ± 0.02	10.2 ± 0.09	9.17 ± 0.04
**Doxorubicin**	1.53 ± 0.02	1.79 ± 0.05	1.85 ± 0.01	1.76 ± 0.01	1.50 ± 0.02	1.69 ± 0.03

*^a^* HCT-15, colon cancer; NCI-H23, lung cancer; ACHN, renal cancer; MDA-MB-231, breast cancer; NUGC-3, stomach cancer; PC-3, prostate cancer; *^b^* GI_50_ values are the concentrations corresponding to 50% growth inhibition. Data are an average of at least two tests.

## 3. Experimental Section

### 3.1. General Experimental Procedure

The optical rotations were measured using a JASCO digital polarimeter using a 5 cm cell. IR spectra were recorded on a JASCO FT/IR-4100 (Jasco Inc., Easton, MD, USA). ^1^H-NMR spectra were recorded on a Varian Unity 500 (500 MHz) spectrometer (Varian Inc., Palo Alto, CA, USA). Chemical shifts are reported in ppm from tetramethylsilane with the solvent resonance resulting from incomplete deuteration as the internal references (CDCl_3_: δ_H_ 7.26 ppm). ^13^C-NMR spectra were recorded on a Varian Unity 500 (125 MHz) spectrometer with complete proton decoupling. Chemical shifts are reported in ppm from tetramethylsilane with the solvent as the internal reference (CDCl_3_: δ_C_ 77.26 ppm). HPLC was performed with YMC-Pack Silica columns using a Shodex RI-101 detector (Showa Denko K.K., Tokyo, Japan).

### 3.2. Biological Material

The sponge *Scalarispongia* sp. was collected by hand using SCUBA at a 10 m depth offshore of Dokdo (island), Republic of Korea. The growth form of this specimen was a flattened or plate-like structure, up to 70 mm in size and 10 mm in thickness; the color in ethanol was black externally and light brown internally. The specimen’s surface was uneven and unarmored, with microconules (1–2 mm) that are formed by protruding fibers scattered over the surface. The skeleton was of very wide regularity. Furthermore, it was reticulate in appearance (with prominent ladder-like shapes), consisting of primary (150–250 µm) and secondary (80–120 µm) clearly laminated and uncored fibers. The rectangular reticulum of the skeleton net had meshes with width of more than 2000 µm. A certain amount of fasciculation of fibers of both types was observed. The sponge was compressible and not difficult to break. This sponge has some resemblance to *Scalarispongia* (*Porifera*, *Dictyoceratida*, *Thorectidae*) in terms of very regular skeleton organization. The voucher specimens are deposited at the sponge collection of Korea Institute of Ocean Science and Technology (08DD06).

### 3.3. Extraction and Isolation

The collection (1.0 kg, wet wt.) was immediately freeze-dried and kept at −20 °C until the time of our investigation. The sponge was extracted using methanol (1 L × 2) and dicholoromethane (1 L × 1) at room temperature. The combined extract (18.8 g) was partitioned between *n*-butanol and water, and the organic layer (6.4 g) was further partitioned between 15% aqueous methanol and *n*-hexane. The aqueous methanol fraction (4.6 g) partitioned again between dichloromethane and 50% aqueous methanol. Subsequently, dichloromethane fraction (1.7 g) was subjected to a silica gel column chromatography (230–400 mesh, Merck, Hunterdon County, NJ, USA) with ethyl acetate in *n*-hexane (15%, 20%, 30%, 50%, 100% stepped gradient) and 10% MeOH in dichloromethane. The fractions eluted with 20%, 30%, 50%, and 100% ethyl acetate in *n*-hexane were purified by HPLC using silica column to afford **6** (4.8 mg), **3** (9.6 mg), **2** (12.4 mg), **8** (2.7 mg), **7** (1.4 mg), **1** (61.8 mg), **4** (58.4 mg), and **5** (12.9 mg). Each known compound was identified by comparison of ^1^H- and ^13^C-NMR spectra as well as HRMS data with those reported. Compound **5**: pale yellow amorphous solid; [α]^25^_D_ 33.6 (*c* 0.5, CHCl_3_/CH_3_OH (3:1)); UV λ_max_ (log *ε*) 218 (4.05) nm; IR (KBr) ν_max_ 3396, 2941, 1746, 1047, 1047 cm^−^^1^; ^1^H- and ^13^C-NMR (CDCl_3_/CD_3_OD (3:1), 500 and 125 MHz), see Table 1; (+)-HRFABMS *m*/*z* 403.2845 [M + H]^+^ (calcd for C_25_H_3__9_O_4_, 403.2848).

Compound **6**: pale yellow amorphous solid; [α]^25^_D_ 12.3 (*c* 0.5, CHCl_3_); UV λ_max_ (log *ε*) 229 (2.56) nm; IR (KBr) ν_max_ 2928, 1755, 1237, 1067 cm^−^^1^; ^1^H- and ^13^C-NMR (CDCl_3_, 500 and 125 MHz), see Table 1; (+)-HRFABMS *m*/*z* 429.3008 [M + H]^+^ (calcd for C_2__7_H_41_O_4_, 429.3005).

Compound **7**: pale yellow amorphous solid; [α]^25^_D_ 9.2 (*c* 0.5, CHCl_3_); UV λ_max_ (log *ε*) 229 (2.84) nm; IR (KBr) ν_max_ 3400, 2994, 2361, 1765, 1241, 1051 cm^−^^1^; ^1^H- and ^13^C-NMR (CDCl_3_, 500 and 125 MHz), see Table 1; (+)-HRFABMS *m*/*z* 387.2902 [M + H]^+^ (calcd for C_25_H_3__9_O_3_, 387.2899).

Compound **8**: pale yellow amorphous solid; [α]^25^_D_ −7.6 (*c* 0.5, CHCl_3_); UV (MeOH) λ_max_ (log *ε*) 220 (4.04) nm; IR (KBr) ν_max_ 2937, 2360, 1744, 1242, 1045 cm^−^^1^; ^1^H-NMR (CDCl_3_, 500 MHz) δ 6.51 (1H, brs, H-18), 4.99 (1H, brs, H-12), 4.16 (2H, brs, H-19), 2.43 (1H, dd, *J* = 18.0, 3.5 Hz, H-15), 2.34 (1H, dd, *J* = 18.0, 14.0 Hz, H-15), 2.09 (1H, dd, *J* = 14.0, 3.5 Hz, H-14), 2.04 (3H, s, 12-OAc), 1.80 (1H, m, H-11), 1.71 (1H, m, H-11), 1.68 (1H, m, H-7), 1.58 (1H, m, H-1), 1.56 (1H, m, H-2), 1.55 (1H, m, H-6), 1.38 (1H, m, H-6), 1.36 (1H, m, H-2), 1.26 (1H, d, *J* = 13.0, 4.0 Hz, H-9), 1.24 (1H, m, H-3), 1.11 (3H, s, H-24), 1.08 (1H, m, H-3), 0.99 (1H, ddd, *J* = 14.5, 12.5, 3.0, H-7), 0.91 (3H, s, H-23), 0.87 (1H, m, H-5), 0.79 (3H, s, H-21), 0.80 (3H, s, H-22), 0.83 (3H, s, H-20), 0.60 (1H, m, H-1); ^13^C-NMR (CDCl_3_, 125 MHz) δ 202.0 (C-16), 170.8 (12-OAc), 155.3 (C-18), 135.4 (C-17), 76.3 (C-12), 62.1 (C-19), 56.8 (C-5), 52.8 (C-9), 49.4 (C-14), 42.1 (C-3), 41.0 (C-13), 40.7 (C-7), 39.8 (C-1), 37.4 (C-8), 37.1 (C-10), 34.3 (C-15), 33.5 (C-4), 33.5 (C-20), 22.3 (C-11), 21.6 (C-21), 21.5 (12-OAc), 19.6 (C-24), 18.6 (C-6), 18.1 (C-2), 16.6 (C-23), 16.2 (C-22); (+)-HRFABMS *m/z* 417.3008 [M + H]^+^ (calcd for C_2__6_H_41_O_4_, 417.3005).

### 3.4. Cytotoxicity Assay

The growth inhibition assays against human cancer cell lines, in particular, HCT-15 (colon), NCI-H23 (lung), ACHN (renal), MDA-MB0231 (breast), NUGC (stomach) and PC-3 (prostate), were carried out according to a published protocol [23]. In brief, cancer cells were added to a 96-well plate containing control (doxorubicin) or test compounds. After being incubated for 48 h, cultures were fixed with 50% trichloroacetic acid (50 μg/mL) and stained with 0.4% sulforhodamine B in 1% acetic acid. Unbound dye was removed by washing with 1% acetic acid and protein-bound dye was extracted with 10 mM Tris base (pH 10.5) for determination of optical density. The absorbance at 540 nm was determined using a VersaMax microplate reader (Molecular Devices, LLC., Sunnyvale, CA, USA).

## 4. Conclusions

In summary, eight scalarane sesterterpenoids (**1**‒**8**), including four new compounds (**5**‒**8**), were isolated from the Korean marine sponge of *Scalarispongia* species. Among the new compounds, compounds **6** and **7** are the first isoscalarane derivatives lacking the C-12 oxygen, and compound **8** is diametrically opposed to other reported tetracyclic scalaranes, which have an oxo group at C-19 and a hydroxy or an acetate group at C-16. The isolated compounds showed cytotoxicity against a panel of human cancer cell lines in all tested cases, with the exception of compound **5**, which showed no cytotoxicity against the cancer cell lines considered in this study. The results presented in this contribution may provide new insight into the mode of cytotoxicity of scalarane sesterterpenoids.

## Supplementary Materials

Supplementary materials can be found at http://www.mdpi.com/1422-0067/15/11/20045/s1.
